# A Ratiometric Wavelength Measurement Based on a Silicon-on-Insulator Directional Coupler Integrated Device

**DOI:** 10.3390/s150921280

**Published:** 2015-08-28

**Authors:** Pengfei Wang, Agus Muhamad Hatta, Haoyu Zhao, Jie Zheng, Gerald Farrell, Gilberto Brambilla

**Affiliations:** 1 Photonic Research Centre, Dublin Institute of Technology, Kevin Street, Dublin 8, Ireland; E-Mail: gerald.farrell@dit.ie; 2 Optoelectronics Research Centre, University of Southampton, Southampton SO17 1BJ, UK; E-Mail: gb2@orc.soton.ac.uk; 3 Department of Engineering Physics, Faculty of Industrial Technology, Institut Teknologi Sepuluh Nopember, Surabaya, Sukolilo 60111, Indonesia; 4 Key laboratory of In-fiber Integrated Optics of Ministry of Education, College of Science, Harbin Engineering University, Harbin 150001, China; E-Mail: zhaoyu1314522@163.com; 5 State Key Laboratory on Integrated Optoelectronics, College of Electronic Science and Engineering, Jilin University, Changchun 130012, China; E-Mail: zhengjie@jlu.edu.cn

**Keywords:** directional coupler, wavelength monitor, integrated optics

## Abstract

A ratiometric wavelength measurement based on a Silicon-on-Insulator (SOI) integrated device is proposed and designed, which consists of directional couplers acting as two edge filters with opposite spectral responses. The optimal separation distance between two parallel silicon waveguides and the interaction length of the directional coupler are designed to meet the desired spectral response by using local supermodes. The wavelength discrimination ability of the designed ratiometric structure is demonstrated by a beam propagation method numerically and then is verified experimentally. The experimental results have shown a general agreement with the theoretical models. The ratiometric wavelength system demonstrates a resolution of better than 50 pm at a wavelength around 1550 nm with ease of assembly and calibration.

## 1. Introduction

The measurement of an unknown optical wavelength is a common measurement for many systems, either for test purposes or as an integral part of the operation of a system. Examples include the measurement of wavelength in a multichannel Dense Wavelength Division Multiplexing (DWDM) optical communication system, Fibre Bragg Grating (FBG) based optical sensing system and the estimation of the wavelength of lasers or tunable lasers during the manufacturing process. For a DWDM optical communication system, wavelength measurement is indispensable in the accurate setting and maintaining of the transmitter’s or of the monitoring tunable lasers’ wavelength. For FBG based optical sensing systems, a cost-effective wavelength measurement scheme is very important in their successful commercialisation [1].

The existing numerous wavelength measurement schemes can be divided into two main schemes, namely passive and active wavelength measurement schemes. Most of the existing passive schemes, employing wavelength sensitive optical devices, have a simple configuration and offer high-speed measurements, but suffer from a limited resolution due to associated problems with the use of bulk-optic filter/collimation components and associated alignment stability or a limited wavelength range due to the spectral characteristics of the employed optical devices. The active schemes, mainly using wavelength-scanning technologies, which can achieve high resolutions, require much more complicated configurations and have a low measurement speed as compared to the passive schemes. The classic commercial instruments measuring the wavelength of light involves an interferometer or a monochromator, suffering from high cost and inherently slow measurement speed due to the mechanical motion involved.

A passive ratiometric wavelength monitor [2] offers a high-speed wavelength measurement with a smart footprint and a simple configuration. It consists of a splitter connected to an edge filter and a reference arm. The wavelength of the input signal is determined by the measurement of the ratio of the signal intensities. A ratiometric wavelength measurement scheme can be implemented with bulk devices, either an all-fibre based configuration [3] or integrated optical circuits [4]. Compared to bulk optical devices, integrated wavelength monitors have a low fabrication cost, compact size, high scalability and also benefit from a fast response and physical robustness. Examples include a multimode interference coupler, a system comprising three single-mode rectangular waveguides, a Y-branch with an S-bend structure, and a Y-branch with a multimode interference (MMI) structure [5].

The directional coupler is a fundamental photonic building block which has been commonly used as power dividers [6], optical switches [7], filters [8] and modulators [9] in telecommunication applications. To date some research has been undertaken on the use of a directional coupler structure as an edge filter for use as wavelength monitor [10]. A directional coupler-based edge filter for wavelength monitoring can be directly designed from the waveguide parameters (geometry and refractive index profile) by calculating the spectral response of the directional coupler over the desired measurable wavelength range for each different interaction length with a given separation distance, *i.e.*, by scanning the parameter-space (consisting of the separation distance and interaction length) in the 3-D case within a specified wavelength range, as shown in [11]. However, this method is time consuming, and also it only considers the central coupling region, whereas a complete planar lightwave circuit (PLC) implementation of a directional coupler should consist of an input waveguide region, a central coupling region and an output waveguide region. Therefore the entire PLC structure needs to be considered to take into account the couplings in the input and output waveguide regions.

Silicon-on-insulator (SOI) is an attractive platform for dense integration of optoelectronic devices, and a low-cost integrated optoelectronic circuit on SOI can be realized because the fabrication technology of optical waveguide devices based on SOI technique is compatible with mature CMOS technology [12]. Traditionally SOI consists of a thin silicon layer on top of an oxide cladding layer carried on a bare silicon wafer. With its silicon core and its oxide cladding, it has a high vertical refractive index contrast for better confining the fundamental guided modes in the silicon core. In addition, both the silicon and the oxide (normally silica) are transparent at telecom wavelengths of 1.3 and 1.55 μm. Therefore it can be a potential candidate to develop an SOI based integrated photonic device for telecom applications.

In this paper a simple design method is proposed (Section 2) to determine the required separation distance and interaction length of an SOI directional coupler to meet a desired spectral response as an edge filter device within an integrated wavelength monitoring system. The directional coupler design parameters, namely separation distance and interaction length, are solved with much less computation as compared to the parameter-scanning method described above in [11]. A numerical example is presented in Section 3 using a traditional beam propagation method (BPM) and the experimental verification is also presented in Section 4. In Section 5, a ratiometric wavelength measurement system using the fabricated directional coupler SOI integrated device as an edge filter is presented.

## 2. Theoretical Design

**Figure 1:**
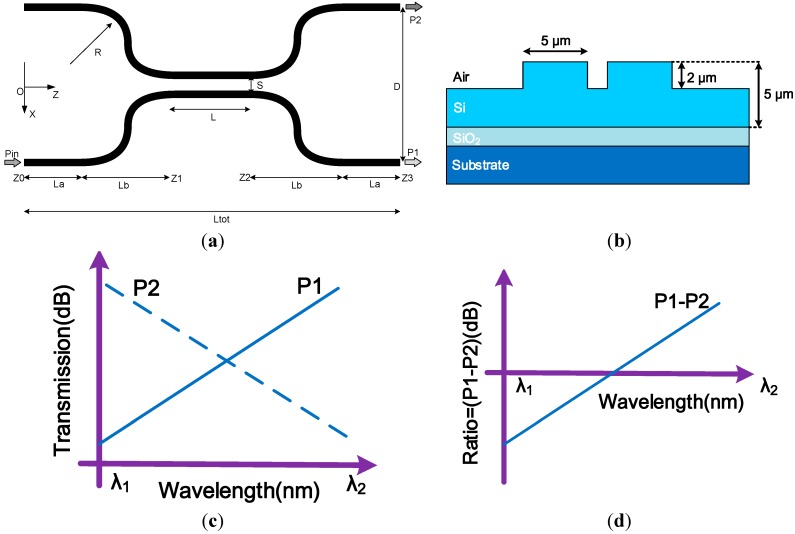
(**a**) Schematic structure of an integrated ratiometric wavelength monitoring based on a directional coupler consisting of an input region, a central region and an output region; (**b**) schematic cross section of SOI waveguide directional coupler; (**c**) desired spectral response; (**d**) output ratio of the two arms over the wavelength range.

The schematic of a simple ratiometric structure based on a directional coupler is shown in Figure 1 and consists of an input waveguide region, a central coupling region and an output waveguide region. Two important parameters define the device: The interaction length (*L*) of two parallel waveguides and their separation distance (*S*) in the central region. For the input and output waveguide region, the separation between the two parallel waveguides increases gradually to 250 μm to allow connecting the directional coupler integrated device with the external coupling waveguides, normally standard singlemode optical fibres. The schematic cross section of the SOI integrated device is shown in Figure 1b. The desired spectral response as the ratio of the output power from the two arms of the parallel waveguides is presented in Figure 1c and the corresponding ratio of the two outputs over the wavelength range is presented in Figure 1d. The wavelength of an unknown input signal can be determined by measuring the power ratio between the two output arms as shown in Figure 1a, assuming a suitable calibration has taken place.

A procedure to design the ratiometric wavelength monitor based on a directional coupler has been presented in our previous work [13] and it is adapted in this formulation. The relative output powers of the directional coupler according to an analysis of the local supermodes [11] can be given by:

(1a) $$P_{1}=\left(1+\text{cos}\left(\varphi \right)\right)/2$$

(1b) $$P_{2}=\left(1-\text{cos}\left(\varphi \right)\right)/2$$

where $\varphi =\varphi_{in}+\varphi_{c}+\varphi_{out}$ is the total phase difference accumulated in the input region ($\phi_{in}$), in the central coupling region ($\varphi_{c}$) and in the output region ($\varphi_{out}$). In the central coupling region of coupling length *L* (from $z=z_{1}$ to $z=z_{2}$) the phase difference is given by:

(2) $$\varphi_{c}=\pi L/L_{c}$$

where Lc=π(β+−β−) is the coupling length and $\beta_{+}$ and $\beta_{-}$ are the propagation constants of a symmetric and an asymmetric supermodes, respectively. $\beta_{+}$ and $\beta_{-}$ can be calculated using a numerical method such as a finite difference method [11].

In the input and output regions (with a length of $L_{B}$, from $z=z_{0}$ to $z=z_{1}$ and from $z=z_{2}$ to $z=z_{3}$, respectively), the total phase difference can be evaluated using:

(3a) $$\varphi_{in}=\int_{z=z_{0}}^{z=z_{1}}\left[\beta_{+}\left(z\right)-\beta_{-}\left(z\right)\right]dz$$

(3b) $$\varphi_{out}=\int_{z=z_{2}}^{z=z_{3}}\left[\beta_{+}\left(z\right)-\beta_{-}\left(z\right)\right]dz$$

It should be noted that at $z=z_{0}$ and $z=z_{3}$ the coupling between two waveguides is negligible $\left(\beta_{+}=\beta_{-}\right)$, as the distance *D* between the two output waveguides is sufficiently large. Although the finite difference method [14] can be used to calculate $\beta_{+}$ and $\beta_{-}$ at any positions in the input/output region in the *z* direction, it is time consuming. A method which utilizes a beam propagation method (BPM) can be more efficient [15]. As in reference [15], the BPM is used to simulate the light propagation from z = z_0_ to z = z_1_ and a field distribution at z_1_ is obtained. The field profiles of the two supermodes in the central coupling region is calculated by using the FDM. The field distribution at z_1_ and these field profiles are used to calculate the accumulated phase in the input region using a formula from reference [15]. Since the output region is the same as the input, then the accumulated phase is also the same.

**Figure 2:**
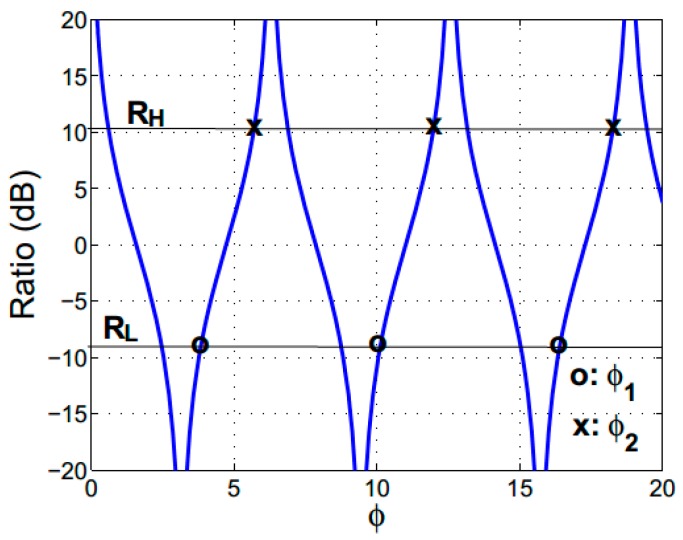
Ratio of output powers as a function of the accumulated phase difference parameter.

The ratio between the two output powers is determined by the accumulated phase difference:

(4) $$Ratio=10\cdot {\text{log}}_{10}\left(P_{1}/P_{2}\right)=10\cdot {\text{log}}_{10}\left(\frac{1+\text{cos}\left(\varphi \right)}{1-\text{cos}\left(\varphi \right)}\right)$$

and it is shown in Figure 2 as a function of the accumulated phase difference. A simple design method was envisaged to determine the separation distance (*S*) and the interaction length (*L*) associated to a specific directional coupler spectral response. The accumulated phase difference can be written as a function of separation distance (*S*) and a wavelength (*λ*) as:

(5) $$\varphi =\varphi_{in}\left(S,\lambda \right)+\varphi_{c}\left(S,L,\lambda \right)+\varphi_{out}\left(S,\lambda \right)=\varphi_{in}\left(S,\lambda \right)+\frac{\pi L}{L_{c}\left(S,\lambda \right)}+\varphi_{out}\left(S,\lambda \right)$$

In the design of the ratiometric wavelength monitor, a chosen range of wavelengths between $\lambda_{1}$ and $\lambda_{2}$ corresponds to the desired ratio from $R_{L}$ to $R_{H}$. Figure 2 shows that values of accumulated phase difference $\varphi_{I}$ and $\varphi_{2}$ for $\lambda_{1}$ and $\lambda_{2}$ correspond to the ratios $R_{L}$ and $R_{H}$, respectively. There are series of $\varphi_{I}$ and $\varphi_{2}$ values as depicted in Figure 2. Insertion of these values of *φ* and *λ*, separately, into Equation (5) produces the two equations:

(6a) $$\varphi_{1}=\varphi_{in,\lambda_{1}}\left(S\right)+\frac{\pi L}{L_{c,\lambda_{1}}\left(S\right)}+\varphi_{out,\lambda_{1}}\left(S\right)$$

(6b) $$\varphi_{2}=\varphi_{in,\lambda_{2}}\left(S\right)+\frac{\pi L}{L_{c,\lambda_{2}}\left(S\right)}+\varphi_{out,\lambda_{2}}\left(S\right)$$

From Equation (6), the elimination of *L* allows to define an objective function *f* in terms of the variable *S* as:

(7) $$f\left(S\right)=\frac{\varphi_{1}-\varphi_{in,\lambda_{1}}\left(S\right)-\varphi_{out,\lambda_{1}}\left(S\right)}{\varphi_{2}-\varphi_{in,\lambda_{2}}\left(S\right)-\varphi_{out,\lambda_{2}}\left(S\right)}-\frac{L_{c,\lambda_{2}}\left(S\right)}{L_{c,\lambda_{1}}\left(S\right)}$$

By using the chosen values of $\varphi_{1}$, $\varphi_{2}$, and utilizing Equation (7), the optimum value of *S* can be obtained which corresponds to *f* (*S*) = 0. Thus, the optimum value of *L* can be extracted from either of Equations (6a) or (6b). Multiple values exist for the parameter pair $\varphi_{1}$ and $\varphi_{2}$, as seen in Figure 2, corresponding to multiple values of *S* and *L*. In practice, the values of $\varphi_{1}$ and $\varphi_{2}$ which yield a viable device size is chosen. And thus, to obtained the desired spectral response, the method proposed here only needs to calculate $L_{c}(S)$ and $\varphi_{in,out}(S)$ for the two wavelengths $\lambda_{1}$ and $\lambda_{2}$ rather than performing spectral response calculation for the whole range of directional coupler parameters in a conventional scanning method.

## 3. Numerical Sample

A numerical example is presented here to demonstrate the design method and the ratiometric scheme. The refractive indices of the core and the cladding for an SOI waveguides are denoted as *n*_co_ and *n*_cl_, respectively. To reduce leakage of the guided mode into the silicon substrate, we chose an oxide thickness of 1 μm. The height and the width of the core were chosen to be 2 and 5 μm respectively, in order to keep a good coupling between the adjacent SOI waveguide and also facilitate modal coupling between the SOI waveguide and the optical fibres. The coupler output was calculated assuming the height (*h*_x_) and width (*h*_y_) of the Si rib waveguide as *h*_x_ = *h*_y_ = 5 μm, *n*_co_ = 3.5, *n*_cl_ = 1 and the curvature radii for the both the input and output waveguides *R*_d_ = 50,000 μm. The calculated wavelength range is from 1500 nm to 1600 nm.

The device is designed to target a discrimination range *R*_1_ = −10 dB to *R*_2_ = 10 dB as depicted in Figure 2. The corresponding accumulated phase difference values are φ_1_ = 3.754 + j2π and φ_2_ = 5.671 + j2π with j = 0, 1, 2, and so on. The procedure given in the previous section provided the separation distance *S* = 1.6, 1.4 and 1.2 um with the corresponding interaction length of *L* = 19,840, 17,540 and 13,640 μm, respectively. The viable device size was chosen from fabrication considerations, to minimise the separation distance and facilitate the deposition process: *S* = 1.6 μm and *L* = 19,840 μm.

**Figure 3:**
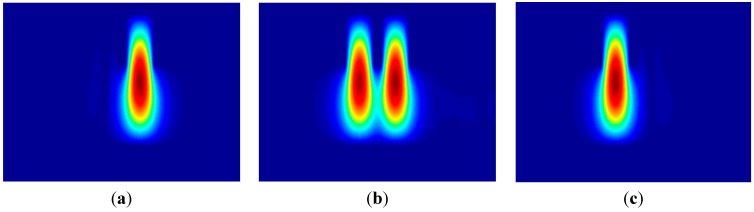
Cross section views of optical field patterns calculated by BPM when output power ratio is (**a**) 1%:99%; (**b**) 50%:50% and (**c**) 99%:1%.

To independently verify the operation of the proposed design, a beam propagation method is used to calculate the whole spectral response with the designed structure within the wavelength range. Figure 3 shows the cross section views of the optical field patterns of the SOI waveguides when the operating wavelength is 1550 nm, for a coupling ratio of 1%:99%, 50%:50% and 99%:1%; the propagation distance are 4720 μm, 5905 μm and 7090 μm, respectively. Figure 3 shows that the coupling has occurred at the bending waveguide region. The accumulated phase difference between the two local supermodes in the input region is then obtained as φ_in_ = 0.13265 (= φ_out_). Also, one can see that the relative output power of the directional coupler varies depending on the propagation distance and hence the coupling ratio varies.

**Figure 4:**
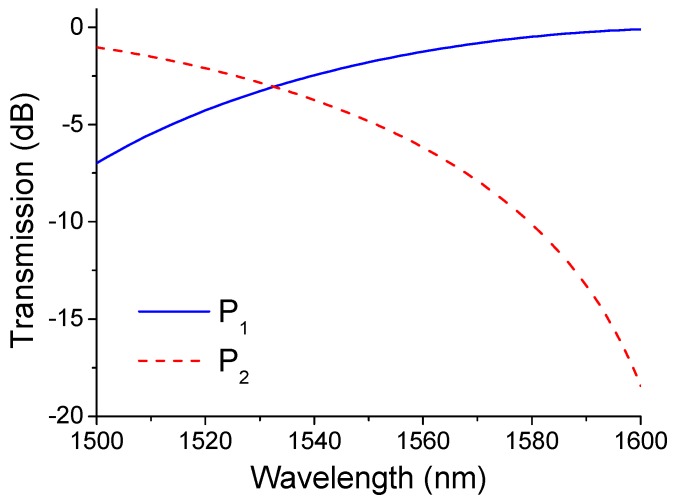
Calculated spectral response of the two arms of the device.

The calculated spectral response is plotted in Figure 4 from which it is clear that the discrimination range (from 1500 to 1600 nm) is −6.99 to −0.1 dB (for P_1_) and −1.03 to −18.44 dB (for P_2_) for the positive and negative slope edge filter, respectively.

## 4. Fabrication of SOI Integrated Device

Devices were patterned on SOI Unibond wafers manufactured by SOITEC with 5 μm thick silicon on a 1 μm thick silica layer. The thick silica layer optically isolates the waveguide from the substrate, reducing losses due to substrate leakage. The wafer was polished on both sidesto reduce the device scattering loss.

**Figure 5:**
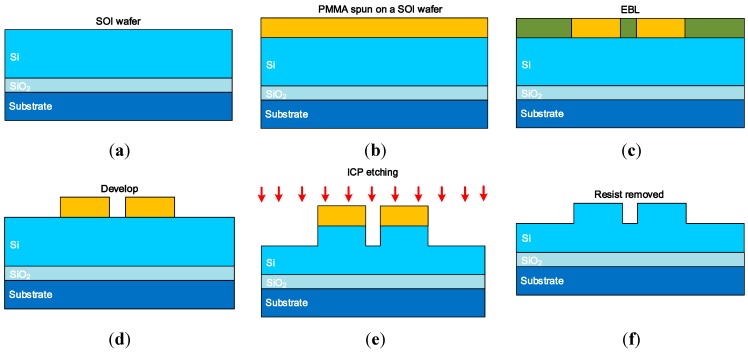
Fabrication process of the SOI waveguide directional coupler, including (**a**) SOI wafer; (**b**) PMMA spun on a SOI wafer; (**c**) EBL; (**d**) Develop; (**e**) ICP etching; (**f**) Photoresist removed.

The entire fabrication process of the SOI integrated directional coupler device is shown in Figure 5. First, the wafer was cleaned by acetone, isopropyl alcohol, methanol and de-ionised (DI) water; then a dehydration baking process was carried out on a hotplate at 120 °C for 3 min. A PMMA based e-beam resist was spun at 6000 rpm on the SOI wafer. The wafer was then prebaked on a hot plate at 180 °C for a minimum of 5 min to vaporize the chlorobenzene and harden the resist, and the resulting PMMA thickness was circa 300 nm. The hardened resist was exposed lithographically using the JBX-9300FS Electron Beam lithography (EBL) system (JEOL, Peabody, MA, USA) with a 20 nm resolution under the accelerate voltage of 10 kV. The electron dose was optimized so that the size of the patterned waveguide was equal to the targeted sizes. The exposed PMMA was developed in a 3:1 mixture of methyl isobutyl ketone (MIBK) and isopropyl alcohol (IPA) for 18 s and subsequently rinsed in IPA for 18 s. The inductively coupled plasma (ICP) etching of the wafer was then carried out with the ICP-RIE (Plasmalab System 100, Oxford Instruments, Abingdon, UK) system with an etch rate of circa 200 nm/min. The entire etching condition was carefully optimized to achieve a minimum sidewall roughness. After the removal of photoresist in O_2_ plasma, the devices were cleaned and cut from the wafer and the devices were polished on both sides for measurements.

**Figure 6:**
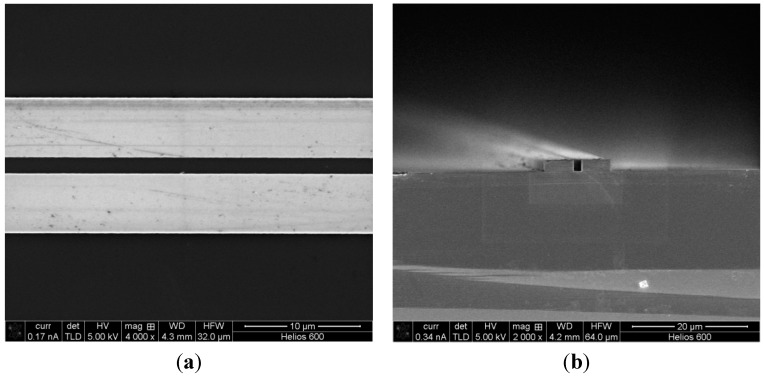
SEM images of the fabricated SOI integrated device: (**a**) top view of the parallel waveguides; (**b**) cross section view of the parallel waveguides.

The SEM images of the coupling region of the fabricated SOI device are shown in Figure 6. The figures have shown both a top view and a cross sectional view of the resulting dry etched features, indicating sidewall verticality and etching surface quality of the waveguides. The Plasmalab System 100 clearly demonstrated the capability of etching high quality SOI integrated photonic structures.

## 5. Characterization of Fabricated Sample

**Figure 7:**
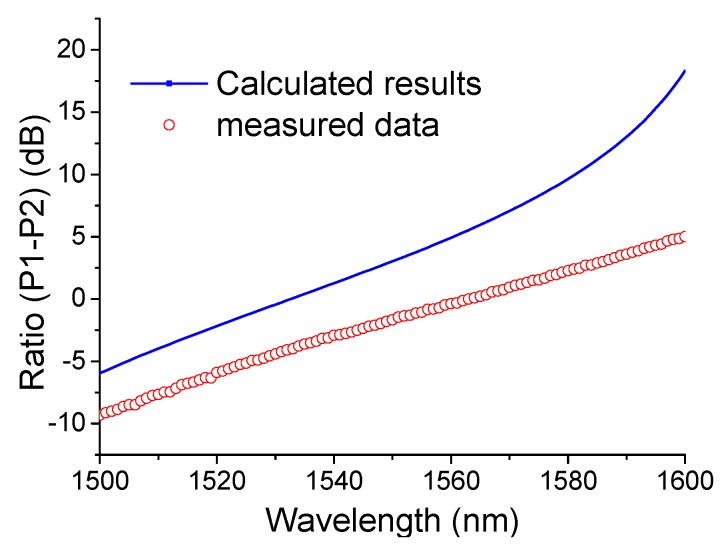
Calculated and measured power ratio results of the fabricated SOI integrated device.

The optical characterization was performed using a tunable laser coupled to the SOI integrated device via a polarization maintaining tapered fibre and a polarization controller. The output light from two arms was collected separately using two 20× objective lenses and two photodiodes placed at the ends of each arm to measure the optical power and hence the power ratio of the system. Figure 7 shows the measured and simulated ratio results. The measured ratio data has a general agreement with the calculated results. From Figure 7 one can see the measured results of the SOI integrated device are smaller than that for the device as predicted by the theoretical calculation. We note a circa 40.96% discrepancy existing on the discrimination range between the measured data and the simulated results, which are consistent on two different setups, thus pointing to either an imprecise refractive index used in the simulations, or more likely, variations in the physical dimensions of the parallel waveguides, the gap between the parallel waveguides and the slab thickness of the SOI, namely the manufacturing tolerances.

**Figure 8:**
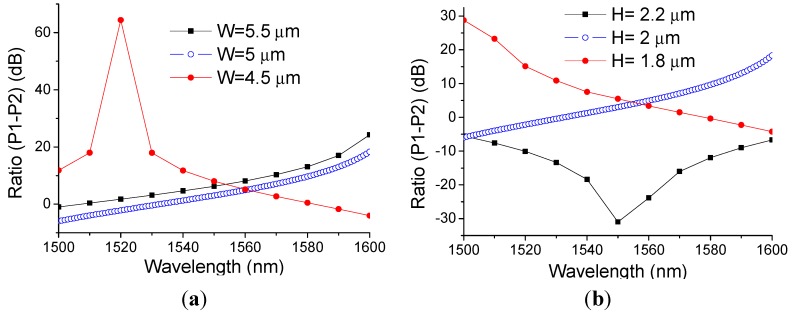
Ratio response of the designed structure with disturbed parameters: (**a**) W = 5 ± 0.5 μm; (**b**) H = 2 ± 0.2 μm.

In order to show the influence made by the manufacturing tolerances, as an example we calculated the ratio of the SOI directional coupler structure with a disturbance to the optimised parameters. Corresponding calculated results are presented in the Figure 8a,b. A disturbance of ±10% to the width and the height of parallel waveguides leads to a maximal variation of 64.85 dB at the wavelength of 1520 nm as shown in Figure 8a. From these simulated results, one can see that the fabricated samples are close to the optimized parameters in the theoretical design.

Compared with the slope of 40 dB/nm over a wavelength range of 1.2 nm presented in [16], the work presented in this paper is 0.15 dB/nm over a wavelength range of 100 nm. Therefore this integrated photonic device can be used for a coarse wavelength measurement system over a broad wavelength range.

**Figure 9:**
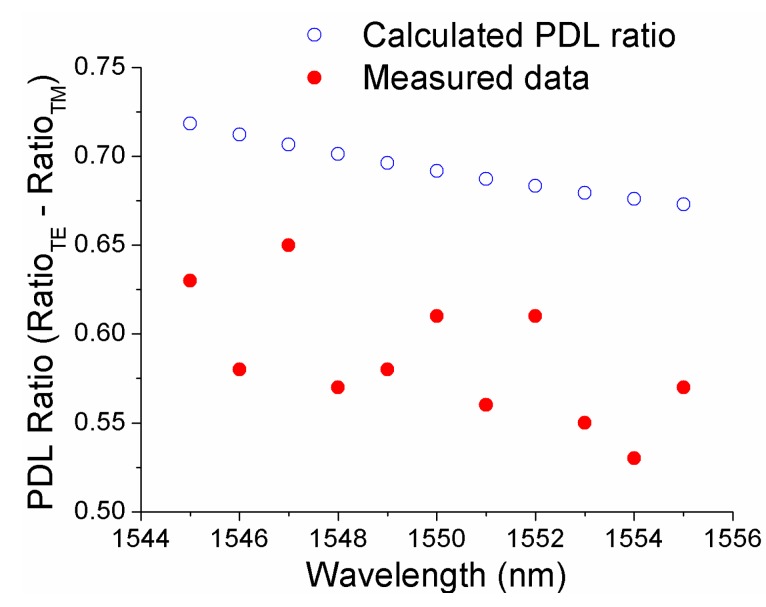
Calculated and measured PDL ratio.

It is well known that the SOI-based photonic devices have a polarization dependent loss (PDL) and a temperature dependence loss (TDL) [17,18]. Both PDL and TDL can reduce the accuracy of wavelength measurement. The PDL was calculated for the ratio (P1-P2) in both quasi transverse electric (TE) and quasi transverse magnetic (TM) modes using the above methods [17]. As shown in Figure 9, the calculated PDL ratio Ratio_PDL_ = |Ratio_TE_ – Ratio_TM_|, for the selected wavelengths of 1545, 1550, and 1555 nm are 0.7148, 0.6917 and 0.6729 dB, respectively. The measured PDL ratio results have a general agreement with the modelled data. Both the PDL and TDL induced wavelength measurement errors are being currently investigated and the results will be reported in due course.

## 6. Ratiometric Wavelength Measurement

Resolution for a ratiometric wavelength measurement system, *i.e.*, the minimum wavelength shift it can detect in a range of practical applications, is an important performance parameter when the system is particularly used for monitoring a tunable laser or in an FBG based sensing system. According to the previous theoretical and experimental investigation [19] of the transmission response of an edge filter in a wavelength measurement application, resolution is restricted by a number of impact factors in the measurement system, including the slope of the transmission response of the edge filter, the measurable power range of the photodetector, the signal-to-noise ratio (SNR) of the optical source. In order to demonstrate the resolution of the wavelength measurement system based on the designed SOI directional coupler structure, the ratiometric wavelength measurement is performed by taking into account the above impact factors. The source wavelength is changed continuously around 1550 nm with a successive interval of 50 pm. For each tuned wavelength, the photodetectors outputs are sampled 100 times and the ratio of the photodetectors outputs is measured. The wavelength is incremented again and the process of sampling is repeated. The complete time series of the measured ratio values as a function of sample time is shown in Figure 8. From Figure 8, it is clear that the detectable ratio due to the wavelength tuning has a potential resolution at least equal to 50 pm, using the SOI directional coupler structure designed in this paper.

How to achieve a high wavelength resolution is a key issue for the ratiometric wavelength system. Intuitively a straightforward view is that the higher the slope of the edge filter, the higher the ratio slope system. However, in practice it is found that this is not the case. The maximum slope of the ratiometric system is determined by not only the slope of the edge filter, but also by the SNR of the input optical signal and the noise of photodetectors, along with the working wavelength range of the system. In practice, the following methods can be utilized to achieve a maximum resolution for the ratiometric wavelength measurement system: (1) a laser source with a higher SNR and narrower bandwidth mean a better system ratio performance; (2) the choice of the edge filter slope needs to be based on a compromise between achieving the highest resolution and the widest wavelength range; (3) another simple way to improve the resolution of the wavelength measurement system is to suppress the fluctuation, e.g., using signal averaging or filtering techniques.

**Figure 10:**
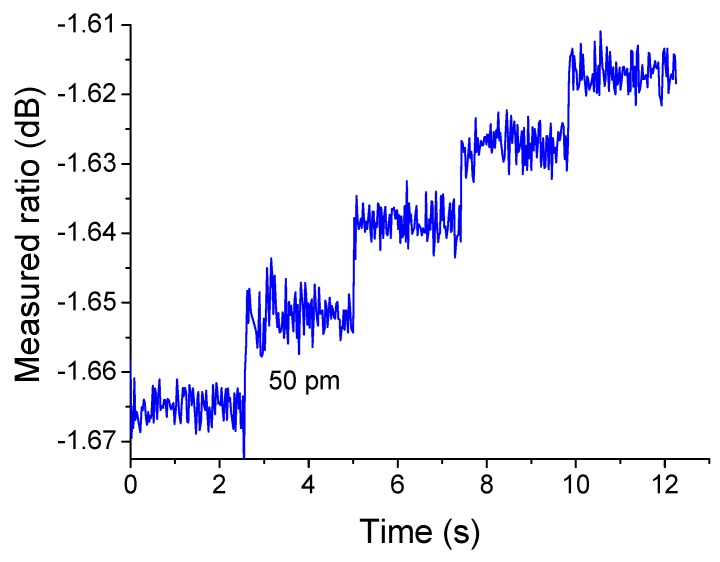
Measured output of the ratiometric system as the input signal increases by 50 pm around the wavelength of 1550 nm.

As shown in Figure 10, the wavelength is stepped by 50 pm every 2.5 s and one can also see that the clearly detectable change of the recorded output of the system shows a resolution of better than 50 pm, which is competitive as compared with some active wavelength scanning techniques, with the advantages of robustness and no mechanical movement. Also compared our previous work on optical fibre based edge filters [3,20], the advantage of this work is that silicon photonics is a new technology that should at least enable electronics and optics to be integrated on the same optoelectronic circuit chip, leading to the production of low-cost devices on silicon wafers by using standard processes from the microelectronics industry. Also the next generation of optical components needs to be low cost and compatible with high-volume manufacturing. Silicon photonics, using highly confined optical modes in silicon waveguide, appears as a unique opportunity to cope with this integration challenge.

## 7. Conclusions

A simple integrated ratiometric wavelength monitor based on an SOI directional coupler integrated device has been designed theoretically and experimentally fabricated. The separation distance and interaction length of the SOI device are calculated according to the desired spectral response. The wavelength discrimination of the designed directional coupler structure has been demonstrated numerically and experimentally, and a ratiometric wavelength measurement has been also undertaken, a competitive resolution better than 50 pm for a rapid passive wavelength measurement with the advantages, such as a simpler configuration and low-cost has been shown as compared to existing ratiometric wavelength measurement systems.
